# Modulators of HIF1α and NFkB in Cancer Treatment: Is it a Rational Approach for Controlling Malignant Progression?

**DOI:** 10.3389/fphar.2013.00013

**Published:** 2013-02-12

**Authors:** Marco Tafani, Bruna Pucci, Andrea Russo, Luana Schito, Laura Pellegrini, Giulietta A. Perrone, Lidia Villanova, Luisa Salvatori, Linda Ravenna, Elisa Petrangeli, Matteo A. Russo

**Affiliations:** ^1^Department of Experimental Medicine, Sapienza University of RomeRome, Italy; ^2^Laboratory of Molecular and Cellular Pathology – Istituto di Ricovero e Cura a Carattere Scientifico San Raffaele PisanaRome, Italy; ^3^Department of Surgical Pathology, Istituti di Ricovero e Cura a Carattere Scientifico Istituto Regina ElenaRome, Italy; ^4^Vascular Program, Institute of Cellular Engineering, Johns Hopkins University School of MedicineBaltimore, MD, USA; ^5^John A. Burns School of Medicine, University of Hawaii Cancer Center, University of HawaiiHonolulu, HI, USA; ^6^UOC of Pathologic Anatomy, San Filippo Neri HospitalRome, Italy; ^7^Division of Endocrinology, Gerontology, and Metabolism, Department of Medicine, Stanford UniversityStanford, CA, USA; ^8^Institute of Molecular Biology and Pathology, Consiglio Nazionale delle RicercheRome, Italy

**Keywords:** hypoxia, inflammation, cancer, HIF1α inhibitors, NFkB inhibitors, sirtuin activators

## Abstract

HIF1α and NFkB are two transcription factors very frequently activated in tumors and involved in tumor growth, progression, and resistance to chemotherapy. In fact, HIF1α and NFkB together regulate transcription of over a thousand genes that, in turn, control vital cellular processes such as adaptation to the hypoxia, metabolic reprograming, inflammatory reparative response, extracellular matrix digestion, migration and invasion, adhesion, etc. Because of this wide involvement they could control in an integrated manner the origin of the malignant phenotype. Interestingly, hypoxia and inflammation have been sequentially bridged in tumors by the discovery that alarmin receptors genes such as RAGE, P2X7, and some TLRs, are activated by HIF1α; and that, in turn, alarmin receptors strongly activate NFkB and proinflammatory gene expression, evidencing all the hallmarks of the malignant phenotype. Recently, a large number of drugs have been identified that inhibit one or both transcription factors with promising results in terms of controlling tumor progression. In addition, many of these molecules are natural compounds or off-label drugs already used to cure other pathologies. Some of them are undergoing clinical trials and soon they will be used alone or in combination with standard anti-tumoral agents to achieve a better treatment of tumors with reduction of metastasis formation and, more importantly, with a net increase in survival. This review highlights the central role of HIF1α activated in hypoxic regions of the tumor, of NFkB activation and proinflammatory gene expression in transformed cells to understand their progression toward malignancy. Different molecules and strategies to inhibit these transcription factors will be reviewed. Finally, the central role of a new class of deacetylases called Sirtuins in regulating HIF1α and NFkB activity will be outlined.

## Introduction: A New Paradigm on Cancer Pathogenesis

The currently prevalent theory on cancer pathogenesis still assumes that the basic events for carcinogenesis and metastasis are mutations that are accumulated by a single cell during its life. Although Weinberg demonstrated that a combination of mutated oncogenes and/or suppressor genes transfected in a normal cell can produce a fully transformed cell, there is no demonstration that another set of genes can produce the malignant phenotype, invading and forming metastasis.

Hanahan and Weinberg (2011) proposed that microenvironment could participate to the progression in many ways: providing VEGF, cytokines, and other growth and survival factors, mostly from activated mesenchymal and inflammatory cells; and creating a reactive oxygen species (ROS)-rich microenvironment which could favor new mutations.

### Mutations for transformation

*Transformation* is characterized by loss of control of proliferation and/or of apoptosis and it is due to an accumulation of mutations with gain-of-function (oncogenes) or loss-of-function (oncosuppressor genes) of gene families related to the cell cycle control and apoptosis control (Hanahan and Weinberg, 2011; Larsson, 2011). Mutations of genes belonging to the DNA repair mechanisms may be responsible for the upstream steps in this sequence, increasing the chances of accumulation of the mutations needed to have a *transformed* phenotype. Experimental models of transformation definitely have established the cause/effect relationship between certain mutations of these genes and the precise generation of a transformed phenotype (Elenbaas et al., 2001; Ince et al., 2007).

Genomic instability, chemical mutagens, and radiations are responsible for random mutations that can involve transformation-related genes. Epigenetic changes (methylation or acetylation status of DNA) and alterations in chromatin structure maintenance mechanisms can stably achieve biological effects (gain-of-function, loss-of-function) on proliferation and apoptosis control, similar to the classical mutations (Huang et al., 2011; Vanden Berghe, 2012). Mutations can be established in somatic (stem) cells in any period of life or can be present in the zygote being inherited from parents. This last type of mutations is responsible for *inherited* *tumor risk* and usually regards a loss-of-function of oncosuppressor genes. A gain-of-function of oncogenes (such as *ret* oncogene) can be observed only exceptionally (Traugott and Moley, 2010). This can be explained by the fact that the presence of oncogene mutations, disrupting normal morphogenesis and development, lead to premature embryonic or fetal death.

### Adaptational responses for progression

*Progression* is characterized by the acquisition of the malignant phenotype that leads to a clinically significant tumor. Malignancy includes ability to grow above the limited dimensions conditioned by diffusion of oxygen and nutrients in the absence of newly formed vessels (neoangiogenesis), ability to extrude and/or inactivate entire families of molecules (resistance to drugs), invasion of adjacent tissues (degradation of BM and ECM), ability to detach from original tissue (changes in adhesive molecules and properties), to migrate in response to a chemoattractant (receptors for chemokines and other chemoattractants), to homing in a specific site that will harbor the new tumor (expression of new sets of adhesive molecules which will encounter their countereceptors on an otherwise activated distant endothelium; Furuta et al., 2010; Zigler et al., 2010; Noman et al., 2011; Nasr and Pelletier, 2012). Most of these genes have been individually studied and analyzed for their mutation, epigenetic changes, and other abnormalities to determine their contributes to the malignancy.

However, the understanding of the progression and all the properties of a malignant cell in terms of mutations of all the necessary genes, has been disappointing and unrealistic. These genes are so numerous that the stochastical occurrence of their mutations during the entire human life is statistically improbable or impossible. Today it is generally accepted that, although mutations of progression-related genes may contribute to the malignancy, other factors, not necessarily mutations, are responsible for the pathogenetic sequence leading to the malignant phenotype.

In the last decade the tissue environment in which the tumor originate and manifest has been subjected to an intensive study. Results of this analysis show that microenvironment of both host tissue and tumor tissue contributes in many ways to the progression and to the final destination of a tumor. A plethora of papers have shown that the contribution depends on cell involved, on local interaction among cells, on paracrine signals generated, on the level of local hypoxia, on the presence of an active local immune-inflammatory response with activated leukocytes and on many other factors (Zigler et al., 2010; Noman et al., 2011; Coleman et al., 2012; Hanahan and Coussens, 2012; Hao et al., 2012; Mucaj et al., 2012; Muratori and Tamagnone, 2012; Nasr and Pelletier, 2012).

Among these so many different contributions, it is difficult to evaluate the precise role of each factor as well as their position in the pathway originating a malignant tumor. In addition, they are too heterogeneous to be included in a logical and sequential pathway in the attempt to fully explain the various facets of the malignant phenotype. Any available unitary framework, including the one recently proposed by Hanahan and Weinberg (2011), is unable to contain all the heterogeneous observations and experiments.

However, papers from many laboratories converged toward an unitary explanation of the progression of the early transformed cells. From one side it has been demonstrated that a great promotion to the malignancy can come from gene adaptation to the hypoxia (Shay and Celeste Simon, 2012). From the other side it has been shown that many proinflammatory genes are overexpressed by malignant cells (Tafani et al., 2011a,b; Jin et al., 2012; Schito et al., 2012; Yao et al., 2012; De Santis et al., 2013). Bridging hypoxia adaptation and proinflammatory gene expression by cancer cells, we suggested the hypothesis that these two very complex cell responses, when sequentially activated, could be a good candidate framework to explain all the properties of the malignant phenotype. In addition, we suggested that transformed (still non-progressed) tumor stem cells could best adapt to generate this kind of responses.

In the next paragraph we will analyze the molecular and biological effects of the hypoxia on transformed cancer cells.

## Hypoxia and Inflammation in Cancer Progression. Generation of a Hypoxic and Proinflammatory Microenvironment in a Growing Early Tumor

Early *transformed* cancer cells are able to proliferate and form small tumors in the absence of neoangiogenesis. Oxygen and nutrients can diffuse from host normal tissue vessels over a radius of no more than 200 μm (Brahimi-Horn et al., 2007). When the small tumor reaches more than 400 μm in diameter, a hypoxic environment is generated, especially in the center of the tumor (Toffoli and Michiels, 2008).

**Figure 1:**
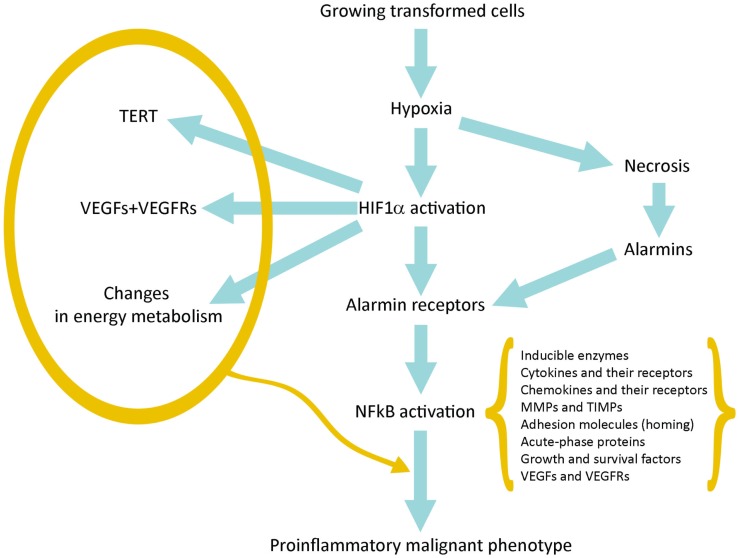
**Schematic representation of the pathway linking hypoxia and HIF1α activation with inflammation and tumor progression**.

Hypoxia produces two basic consequences: (a) *Necrosis* of cells that are more distant from vessels of host tissue; (b) *Activation of HIF1α* in surviving tumor cells closer to the vessels and sublethally damaged; the HIF1α-driven gene expression allows them to survive and grow increasing their commitment to malignancy (Figure 1).

*Activation of HIF1α* leads to the expression of hundreds genes (Table 1). Many of them provide a first impulse (commitment) toward tumor progression. VEGFs and their receptors are responsible for neoangiogenesis and for the possibility to grow above the limit of 400 μm in diameter; telomerase activation increases the proliferative potential and the number of possible cycles; and changes in intermediate and energy metabolism are the best known metabolic effects of this adaptation (Brahimi-Horn et al., 2007; Mucaj et al., 2012).

**Table 1:** **Adaptation to hypoxia in transformed (stem) cells**.

	Genes and molecules activated by HIF1α	Biological adaptation toward malignant phenotype	Reference
1	VRGFs and VEGFRs	Neoangiogenesis	Ahluwalia and Tarnawski (2012)
2	TERT	Increase in telomere length and proliferative potential	Guan et al. (2012)
3	c-Myc; cyclin D1	Increased proliferation	Fer and Melillo (2011), Zhu et al. (2010)
4	TERT, OCT4, Notch	Stem cell renewal	Lee et al. (2012), Qiang et al. (2012)
5	ABC transporter	Drug resistance	Maugeri-Saccà et al. (2011)
6	ALDA, PGK, GLUT-1	Changes in energy metabolism	Mucaj et al. (2012), Semenza et al. (1996), Lam et al. (2009)
7	CXCR4…	Motility	Lu and Kang (2010)
8	MMP9	Integrity of basement membrane, invasivity	Choi et al. (2011b)
9	Alarmin (DAMPs) receptors	NFkB activation; IRR gene expression	Tafani et al. (2011a)

*Necrotic damage* include plasma membrane fragmentation and release of intracellular molecules, some of which constitute alarmins or Damage-Associated Molecular Patterns (DAMPs). The interaction of released alarmins with their receptors triggers a proinflammatory gene expression in various cell types: resident innate immunity cells or leukocytes, which usually express in their plasma membrane a number of alarmin receptors and tumor cells in which alarmin receptors have been induced by hypoxia (Tafani et al., 2011a). Alarmin receptor signaling leads to the activation of NFkB and then to the proinflammatory gene expression. This proinflammatory microenvironment can contribute to tumor progression (see below).

### On the role of HIF1α and HIF1α-dependent genes

By examining the functions of the genes activated by HIF1α it is evident that a number of these genes or gene families play a critical role in pushing a transformed cell toward the acquisition of many hallmarks of malignancy. In particular, the overexpression of VEGFs and their receptors (Ahluwalia and Tarnawski, 2012) activate a tumor-specific neoangiogenesis, allowing the early tumor to grow over the dimensions (200–300 μm), imposed by the simple diffusion of oxygen and nutrients. The activation of telomerase (TERT) increases the length of telomeres and the proliferative potential, immortalizing the involved tumor cells (Guan et al., 2012). A further contribute to the proliferative potential is given by the HIF1α-dependent activation of typical proproliferative genes such as c-myc and cyclin D1 (Zhu et al., 2010; Fer and Melillo, 2011). In addition, HIF1α activates OCT4 and Notch facilitating stem cell renewal, contributing to the immortalization (Lee et al., 2012; Qiang et al., 2012). Resistance to chemotherapy is achieved by overexpression of ABC transporters (Maugeri-Saccà et al., 2011). The overexpression of a number of key-molecules such as ALDA, PGK, GLUT-1, beautifully explains the reprograming of the tumor energy metabolism (increased glucose transport and consumption and high glycolysis with lactate production; Semenza et al., 1996; Lam et al., 2009; Mucaj et al., 2012).

Most of the invasion and metastasis genes are co-controlled by HIF1α and by NFkB. Therefore, they will be examined in the next paragraph.

Finally, more importantly, we have observed that in a hypoxic environment a number of cell types, including cancer and normal stem cells, express *de novo* or overexpress different alarmin receptors (similar to those present in activated leukocytes or CD45+ cells; Tafani et al., 2011a). RAGE, P2X7, TLRs, and others, upon activation by alarmins released by necrotic cells, converge in the activation of NFkB with a robust proinflammatory gene expression (Figure 1). This represents the key event to bridge the adaptation to the hypoxia with the expression of hundreds of genes related to the Inflammatory Reparative Response (IRR) and, very importantly, to the acquisition of classical properties to the malignant phenotype. Table 1 summarizes genes or gene families principally involved in the hypoxia adaptation contributing to the malignant progression. This picture include also the so-called EMT (epithelial-mesenchymal transition), in which all the involved genes can be HIF1α and/or NFkB-dependent (Micalizzi et al., 2010).

### On the role of NFkB and NFkB-dependent genes

Once NFkB has been activated through many different pathways, a complex gene response occurs, with the expression of hundreds of genes belonging to specific gene families including a large number of members functionally related to the inflammatory and reparative response (see Table 2). Individually most of these genes have been implicated in the acquisition of crucial properties of the malignant phenotype, providing a coherent theoretical framework to explain the acquisition of most of the malignant hallmarks as an integrated response and adaptation to the tumor environment.

**Table 2:** **IRR gene expression and malignant phenotype biological properties**.

N	IRR gene families dependent on NFkB	Biological functions leading to malignant phenotype	Reference
1	MMPs and TIMPs	Digestion of basement membrane and ECM; invasion	Tobar et al. (2010)
2	Adhesion molecules and counter-receptors	Detachment; homing; organ/tissue tropism; metastatic pattern	Marcu et al. (2010)
3	Chemokines and their receptors	Migration; homing; metastatic patterns	Lu and Kang (2010)
4	Inducible enzymes (COX2; iNOS)	Extravasation, migration, angiogenesis	Wang and Dubois (2006)
5	Cytokines and their receptors	Local amplification of IRR, proliferation, and survival	DiDonato et al. (2012)
6	VEGFs and VEGFRs	Angiogenesis	Ono (2008)
7	Growth and survival factors	Proliferation; antiapoptosis	Langley and Fidler (2011)
8	Acute-phase proteins	IRR amplification; chemotaxis; repair; DAMPs	Hiratsuka et al. (2008)
9	SOCS	Negative regulation of IRR; antimetastatic	Strebovsky et al. (2012)
10	Nm23	Cytoskeletal regulation and organization	Liu et al. (2011)

#### Inducible enzymes (COX2; 5-LOX, iNOS)

Inducible enzymes produced in activated leukocytes upon activation of NFkB are responsible for mediator molecules production such as prostaglandins, leukotrienes, and NO, leading to the manifestation and amplification of the IRR. Their presence in tumor microenvironment and their expression by tumor cells itself has been one of the earliest observation involving inflammation in the pathogenesis of cancer and its progression (Wang and Dubois, 2006). Molecules produced by these enzymes contribute to the many aspects of tumor progression such as neoangiogenesis, recruitment of leukocyte to the tumor microenvironment, and changes for EMT (Micalizzi et al., 2010). Almost 15 years ago a landmark epidemiological study suggested that the use of low-dose aspirin for cardiovascular prevention drastically reduced the risk for colon cancer (Gustafson-Svärd et al., 1997). These epidemiological observations stimulated a number of other retrospective studies and controlled clinical trials on aspirin and other COX2 inhibitors in preventing tumors and their progression, giving rise to a new era in the understanding the role of inflammation in tumor pathogenesis.

#### Cytokines and their receptors

Cytokines characterizes IRR directly influencing target leukocytes, polarizing the response as Th1 or Th2 and stimulating the proliferation of target cells (CD45+) to reinforce and amplify the IRR (DiDonato et al., 2012). Cytokines are present in most human tumor microenvironment, being produced by cancer cells itself and/or by leukocyte infiltrate (DiDonato et al., 2012). Interestingly, tumor cells express also receptors for various cytokines in parallel with their degree of malignancy (DiDonato et al., 2012). Therefore, thanks to the presence of cytokine receptors, tumor cells can be strongly influenced in their biology, such as proliferation rate (IL-2) and in their polarization (Th1 cytokines) and, probably, in the expression of adhesion molecules and their countereceptors, thus influencing the homing for metastasis (DiDonato et al., 2012).

#### MMPs and TIMPs

MMPs and TIMPs are NFkB-dependent genes normally expressed in activated leukocytes, but it is well known that disruption of the MMP/TIMP activity ratio with a gain-of-function of proteasic activity over basement membrane and extracellular matrix proteins is present in malignant tumors and parallels the invasive potential (Tobar et al., 2010; Choi et al., 2011b). Then the key event for demolishing the physiological tissue barrier (limits) and for invasion to start is basically controlled by both HIF1α and NFkB through the expression of these genes.

#### Adhesion molecules and counter-receptors

The activation of NFkB in leukocytes finely reprograms the expression of adhesion molecules for migration and for homing at constitutive district tissue or at damaged site. A NFkB-dependent and/or cytokine-dependent new expression of adhesive molecules occurs also in tumor cells, allowing a number of biological changes typically related with malignancy. These changes include the ability to detach from the original tissue (i.e., cadherins), the ability to migrate following a specific chemotactic gradient and a path of ECM molecules (receptors for chemokines and integrins), and, finally, the identification of the homing site represented by activated endothelial cells (ICAM-1, selectins, and their countereceptors; Marcu et al., 2010).

#### Chemokines and their receptors

Tumor cells express both chemokines and their receptors in parallel with their degree of malignancy (Lu and Kang, 2010). The production of chemokines give rise to a gradient which is probably the main responsible for the attraction of leukocytes and mononuclear infiltration in advanced tumors (Lu and Kang, 2010). More importantly, the expression of chemokine receptors is a crucial event for the occurrence of metastasis. In fact, metastasis is a complex event which include a number of steps with the participation of hundreds of genes. Detachment from the primary tumor tissue must be followed by a vectorial migration along a chemotactic gradient, which implies the presence of specific receptors for the chemoattractant. CXCR4, a receptor for SDF1α, is the best characterized in tumor cells and has been definitely associated with progression and prediction of metastasis in many human tumors (Lu and Kang, 2010). Both chemokines and their receptors are under the control of NFkB.

#### VEGFs and VEGFRs

The occurrence of a clinically relevant tumor, detectable by the present imaging techniques, needs to grow at the dimension of a few mm in diameter and then, by default, it needs a process of neoangiogenesis, with an adequate expression of VEGFs and VEGFRs in the tumor microenvironment. VGEFs can be produced both by activated leukocytes and mesenchymal cells present in the tumor microenvironment or, more importantly, by tumor cells themselves under the influence of activated HIF1α and NFkB (Ono, 2008). In the last case it has been demonstrated that cancer cells (probably tumor stem cells and progenitors) may express also VEGFRs, suggesting the possibility that tumor cells can contribute to the formation of their new vascular tree (Ono, 2008).

#### Growth and survival factors

HIF1α and NFkB control a number of growth and survival factors and their receptors. This has been demonstrated in activated leukocytes (involved in tissue repair) and in hypoxia-activated tumor cells. This is an additional advantage for tumor growth and a prerequisite for the establishment of a secondary metastatic tumor. The “Seed and soil” hypothesis predicts that a favorable tissue environment is relevant for the occurrence of a metastasis (Langley and Fidler, 2011). In this case growth and survival factors can be provided both by activated leukocytes or mesenchymal cells of the microenvironment and by tumor cells themselves in which proliferative pathways are already activated (transforming oncogenes) or in which these genes are overexpressed upon NFkB activation (Brahimi-Horn et al., 2007).

#### Acute-phase proteins

Acute-phase proteins have been considered plasma markers useful to evaluate the systemic IRR. They include soluble and cell bound isoforms, such as C reactive protein, pentraxin-3, and other pentraxins; their functions are only partially elucidated. Similarly to the other NFkB-dependent genes, they appear expressed or overexpressed in hypoxia-activated tumor cells and in activated leukocytes. Their functions in tumor progression is still debated. From one side, they appear to inhibit tumor cell proliferation and to decrease with progression (Ronca et al., submitted), from the other hand they can be highly expressed in malignant cells compared to the host normal tissue (Hiratsuka et al., 2008).

#### SOCS and negative regulators

NFkB activation include also the expression of a number of negative key-regulator proteins of IRR, such as SOCS-1 (Strebovsky et al., 2012). This latter protein is a member of SOCS family which suppress the cytokine signaling via JAK/STAT, down-regulate TLR expression and signaling and decrease the NFkB activity and duration (Strebovsky et al., 2012). This family and other negative regulators are considered part of the normal feed-back control of the IRR. As predicted by our hypothesis, SOCS-1 decreased in hypoxia-activated cells, as a physiological response of HIF1α-NFkB integrated activation (De Santis et al., 2013).

#### Anti-HIF1α and anti-IRR tumor therapy and cancer prevention

A number of epidemiological studies and some clinical controlled trials support the idea that the negative modulation of IRR reduces the risk and the incidence of some tumors and, in addition, may slow or inhibit their progression toward malignancy. These observations and the experimental studies linking hypoxia, IRR, and tumor progression have suggested new strategies for preventing tumors, reducing their incidence, slowing their progression, substantially increasing survival, and decreasing death ratio for malignancy (Liu et al., 2011).

## Modulators of HIF and HIF-Dependent Genes

**Figure 2:**
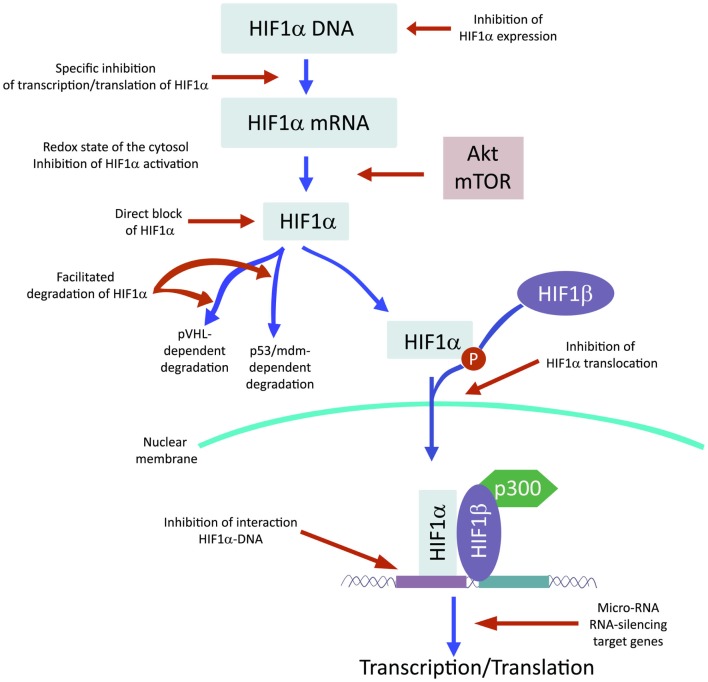
**HIF1α activation can be inhibited at different steps along the pathway**.

**Figure 3:**
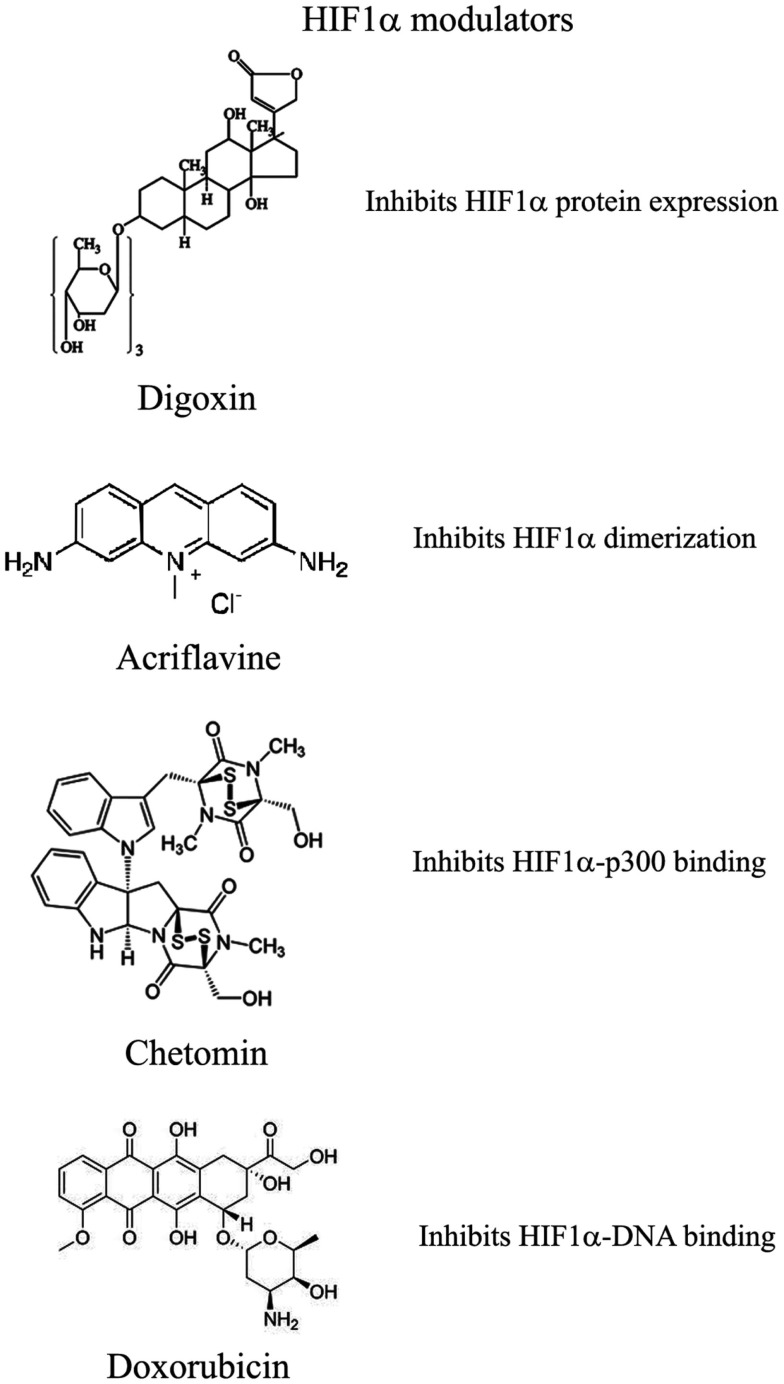
**Selection of compounds that can be used to modulate HIF1α pathway**.

Recently and because of its central role in tumor progression, HIF1α has become the target of an increasing number of inhibitors developed with the aim to block or reduce tumor growth and possibly progression (Semenza, 2012; Xia et al., 2012; Figure 2). However, it must be noted that most of these compounds are FDA approved molecules used for treatment of cancer or other pathologies or natural products and that most of the studies and discoveries on inhibitors or activators of HIF1α have been made in cell-based systems or xenografts by research laboratories and not by pharmaceutical companies (Semenza, 2012). Inhibitors identified so far exert their action through a variety of different mechanisms ranging from decreased mRNA and protein levels of HIF1α, to prevention of HIF1α dimerization or DNA binding, to inhibition of HIF1α binding to co-activators. Some representative compounds acting at different steps of HIF1α pathway are reported on Figure 3.

### Several compounds have been shown to reduce HIF1α protein and mRNA accumulation

Among those: (1) PI3Kinase inhibitors wortmannin and LY294002 as well as mTOR inhibitor rapamycin have been shown to reduce HIF1α protein levels in different cell lines (Jiang et al., 2001; Majumder et al., 2004). In fact, these studies demonstrate that the rapamycin-sensitive functions of mTOR are not essential for the accumulation of HIF1α but are important for full expression of this protein as well as for integrating oxygen and nutrient poor conditions (Majumder et al., 2004). (2) Inhibition of HIF1α protein expression and decreased growth of tumor xenografts has been obtained also with cardiac glycosides such as digoxin, ouabain, etc (Semenza, 2012). Digoxin has been used for treatment of heart disease for a long time and it is therefore of particular interest for a combinatorial therapy with other conventional anti-tumor agents (Zhang et al., 2008). (3) Microtubule targeting agents such as 2 methoxyestradiol (2ME2) and its synthetic derivatives prevents HIF1α translation and nuclear accumulation with a corresponding anti-tumor activity (Mabjeesh et al., 2003). However, the exact mechanism of action of such compounds has not been fully elucidated. (4) Class II histone deacetylase (HDAC) inhibitors such as trichostatin and LAQ824 increased HIF1α ubiquitination and degradation of HIF1α by an unknown mechanism (Qian et al., 2006). The emerging role of class III HDAC on HIF1α stabilization will be discussed in detail below. (5) Locked nucleic acid (LNA)-based oligonucleotides are third generation of antisense technology that offer high stability and long lasting target inhibition. EZN-2968 is a LNA directed against HIF1α that has shown great inhibition of HIF1α and HIF1α-dependent genes and that is currently under phase I clinical study because of its ability to reduce tumor growth in xenografts (Greenberger et al., 2008). (6) Ibuprofen and other NSAIDs decreased HIF1α and HIF2α protein levels in prostate cancer cells by a yet to define mechanism that could involve either the PI3K or the proteasome (Palayoor et al., 2003). However, HIF degradation by ibuprofen was not due to its COX2 inhibition activity (Isaacs et al., 2002). (7) Natural antibiotic geldanamycin (GA) and antifungal radicicol prevent binding of Hsp90 to HIF1α thereby decreasing its stability with subsequent proteasomal degradation (Isaacs et al., 2002). In particular, derivates of GA are now in clinical trials. Similarly, Antimycin A, an antibiotic from *Streptomyces sp*. that induces apoptosis and inhibits the mitochondrial electron transport chain from cytochrome *b* to cytochrome C_1_, decreased HIF1α protein level by an unknown mechanism (Maeda et al., 2006). (8) A plethora of natural products possess inhibitory effects of HIF1α. Many of these substances increase HIF1α degradation by activating proteasomal system or by unknown mechanisms. In particular, moracin O and P derived from *Morus* species (mulberry bark) activates HIF1α degradation (Dat et al., 2009). Other HIF1α inhibitors are manassantin B from the aquatic plant *Saururus cernus* that probably exerts its effect by degrading HIF1α and inhibiting VEGF secretion (Hossain et al., 2005). Curcumin and berberine, derived respectively from the Indian spice turmeric and from the chinese goldthread increases HIF1α proteasomal degradation (Choi et al., 2006). Similar results were obtained with resveratrol, a compound found in grapes and other plants, and with flavonoids such as methylalpinumisoflavone from the tropical legumaceous *Lonchocarpus glabrescens* (Park et al., 2007; Liu et al., 2009). Sibiriquinone A from red sage suppressed HIF1α accumulation and VEGF secretion through HIF1α degradation (Dat et al., 2007). This partial list clearly demonstrates that natural products are an important source of HIF1α inhibitors that act through a variety of different mechanisms many of which still unknown.

### Inhibition of HIF1α dimerization has been demonstrated by two compounds

Acriflavine and Korean red ginseng. Acriflavine, an antibacterial agent, binds to the PAS-B subdomain of HIF1α and HIF2α thereby preventing the binding to HIF1β, an effect that results in reduced VEGF production and tumor growth (Lee et al., 2009). Water extract of red ginseng inhibited HIF1α and 1β dimerization with no toxic effects, however, the mechanism of action and the anti-tumoral effects are not known (Choi et al., 2011a).

### DNA binding of HIF to HRE with expression of HIF1α-dependent genes has been inhibited by several compounds

Synthetic polyamides binding to and inhibiting HRE elements recognized by HIF1α prevented VEGF synthesis (Semenza, 2012). Doxorubicin and daunorubicin bind to DNA and prevent HIF binding, transcription of target genes, and tumor growth (Tanaka et al., 2012). Finally, echinomycin, an antibiotic isolated from *Streptomyces echinatus*, binds to DNA and inhibits HIF1α activity (Wang et al., 2011).

### HIF1α transcriptional activity requires binding to the coactivator p300

Chetomin, a metabolite from the fungus *chaetomium*, prevents HIF-p300 binding by acting on p300 structure and inhibit transcription of HIF target genes (Kung et al., 2004). In nude mice, Chetomin prevented tumor growth without affecting body weight (Kung et al., 2004). Bortezomib, a proteasome inhibitor, binds to the domain of HIF1α that interacts with p300 thereby preventing a functional interaction between these two factors and blocking transcription of target genes (Befani et al., 2012).

## Modulators of NFkB and NFkB-Dependent Genes

**Figure 4:**
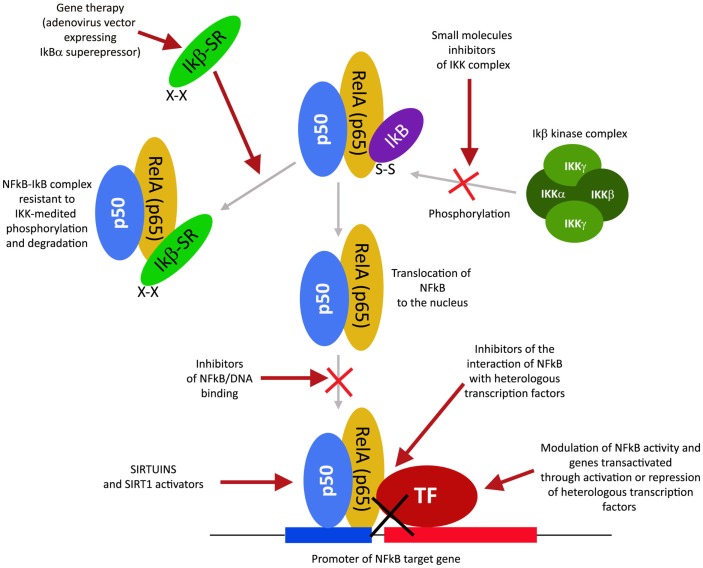
**NFkB activation pathway and its different inhibition steps**.

**Figure 5:**
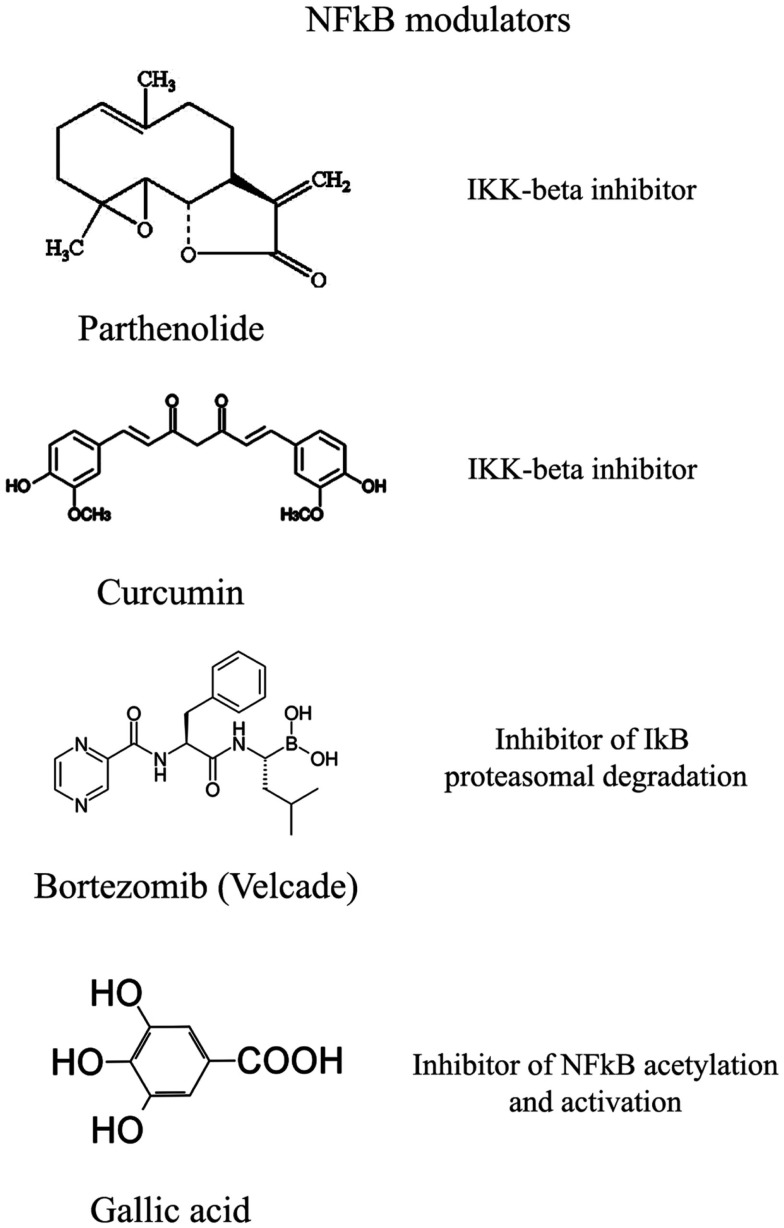
**Selection of compounds that can be used to modulate NFkB pathway**.

The transcription factor NFkB plays a central role during tumorigenesis because promotes the expression of more than 500 genes involved in crucial cellular pathways such as suppression of apoptosis, increased migration and invasion, increased digestion of extracellular matrix, increased expression of adhesion molecules, etc (Gupta et al., 2010; Figure 4). NFkB is regulated by many post-translational modifications such as methylation, acetylation, phosphorylation, ubiquitination. Moreover, once activated, NFkB translocates and accumulates in the nucleus where binds to the DNA and activates transcription of a plethora of genes (Gupta et al., 2010). Therefore, similarly to HIF1α, also NFkB can be modulated by acting on different steps of the pathways that controls its activity. Again, many natural products mostly with anti-inflammatory properties are NFkB inhibitors (Gupta et al., 2010). More than 700 inhibitors of NFkB have been identified so far and their importance is due to the central role that this transcription factor has for many pathologies, beside inflammation and cancer (Wilczynski et al., 2011). Some representative compounds acting at different steps of NFkB pathway are reported on Figure 5.

Moreover, it is possible to group the large number of NFkB inhibitors by considering their mechanism of action. In particular:

### NFkB regulation by protein kinases inhibition or protein phosphatases activation

This class of NFkB inhibitors prevents the activation of IKK kinases. IKK phosphorylates and increases the degradation of the inhibitory subunit IkB, thereby activating NFkB (Gupta et al., 2010). However, only for few IKK inhibitors the mechanism of action is known (Gupta et al., 2010). Among these IKK inhibitors there are natural compounds such as B-carboline (Karin et al., 2004), an indole alkaloid extracted from several plants and that acts as a benzodiazepine agonist, parthenolide, a natural compound isolated from feverfew that acts by interacting with Cys-179 of IKK and, curcumin (Lubbad et al., 2009; Gupta et al., 2010). In particular, parthenolide seems to have an anti-tumoral activity also on cancer stem cells (Gunn et al., 2011). Anti-inflammatory drugs are IKK inhibitors although with an unknown mechanisms (Wilczynski et al., 2011). Among these there are: aspirin, ibuprofen, sulindac, sulfasalazine, and other NSAIDs (Kast, 2006). Other NFkB inhibitors are some kinase inhibitors such as SB203580, PD0980589, tyrosine kinase inhibitors, betaine, etc. (Vanden Berghe et al., 1998). Finally also both IKK dominant negative kinase delivered by adenoviruses and antibodies anti-IKK are alternative strategies that could inhibit NFkB activation (Gupta et al., 2010). By contrast NFkB can be inhibited by activating phosphatases that reduce IkB phosphorylation. For example, cytosine arabinoside dephosphorylates NFKB and induces apoptosis in tumor cells (Sreenivasan et al., 2003).

### NFkB regulation by proteasome inhibition

This strategy exploits the fact that inhibition of IkB degradation by the proteasomal system, results in inhibition of NFkB. A well studied and used proteasome inhibitor is Bortezomib (also mentioned as HIF1α inhibitor) that has been shown to reduce tumor growth in xenograft models and has been successfully used in first-line therapy in combination with other drugs such as cisplatinum (Wilczynski et al., 2011). Inhibition of HIF1α, VEGF, and tumor vascularization by Bortezomib are additional benefits that accompany NFkB inhibition. Other proteasome inhibitors similar to bortezomib include ALLnL, lactacystine, MG132, etc. Finally, second-generation proteasome inhibitors are carfilzomib and salinosporamide that act at nanomolar range, have a lower toxicity and can be delivered orally (Wilczynski et al., 2011; Kale and Moore, 2012).

### NFkB regulation by acetylation inhibition

Similar to phosphorylation by kinases and dephosphorylation by phosphatases, acetylation/deacetylation by acetyl tranferases and deacetylases is considered an equally important post translation modification that control the activity of many proteins (Gray and Teh, 2001). NFkB has been shown to be acetylated on several lysines an event that increase its activation (Kiernan et al., 2003). Therefore, as discussed in major details in the following section, the recent discovery of a new class of deacetylases named Sirtuins has increased the attention toward the possibility to control NFkB activity through these enzymes. In fact, sirtuins activation causes an inhibition of NFkB. Similarly, NFkB can be inhibited by inhibiting acetyl transferases such as p300 and CREB-binding protein (Chen and Greene, 2004). In fact, gallic acid from gallnuts or oak bark and anacardic acid have been shown to inhibit NFkB acetylation and consequent activation (Choi et al., 2009).

### NFkB regulation by inhibition of nuclear accumulation

This approach is based on the fact that preventing NFkB by accumulating in the nucleus also prevents its DNA association and transcription of target genes. However, such mechanism has been documented only for SN50 a peptide with a hydrophobic membrane-translocating region and the NLS of NFkB. SN50 competes with NFkB for the nuclear translocation machinery thereby preventing NFkB nuclear translocation. Unfortunately, SN50 prevents nuclear accumulation of a large number of transcription factors (Sun et al., 2012). A more promising inhibitor is a compound derived from a fungal antibiotic called dehydroxymethylepoxyquinomicin (DHMEQ) that specifically inhibits NFkB nuclear accumulation with anti-inflammatory and anti-tumoral activity (Kozakai et al., 2012).

## Sirtuins and SIRT1 Activators in Modulating HIF1α and NFkB

Sirtuins owe their name to silent information regulator 2 (Sir2), identified in yeast and linked to lifespan extension (Houtkooper et al., 2012). In mammals there are seven Sir2 homologs (SIRTs 1-7). Sirtuins are either class III nicotinamide adenine dinucleotide-(NAD+)-dependent deacetylase or ADP-ribosyl transferases (Houtkooper et al., 2012). Their dependence from NAD+ directly links sirtuins activity to the metabolic state of the cells. For this reason sirtuins have been implicated in many physiological functions such as gene silencing, cell death, longevity, inflammation, and cancer (Houtkooper et al., 2012).

Sirtuins have also shown to associate, deacetylate, and regulate the activity of both HIF1α and NFkB. However, only for SIRT1, 2, 3, and 6 this regulatory function as been demonstrated.

*SIRT1* deacetylates both HIF1α and NFkB. In the case of NFkB all the results so far point toward an inhibition of its signaling following deacetylation by SIRT1 (Morris, 2012). In fact, both *in vitro* and *in vivo* experiments have shown that SIRT1 or activation of SIRT1 by resveratrol and other polyphenols decreases inflammatory response by deacetylating and inhibiting NFkB. These results are particularly interesting considering the central role of NFkB in many cellular pathways involved in inflammation, aging, cancer, etc. Controversial results have been reported, instead, for SIRT1/HIF1α signaling. In fact, it is not yet clear if SIRT1 is influenced or not by hypoxia. Some reports indicates that hypoxia increases SIRT1 levels whereas others that hypoxia decreases SIRT1 (Lim et al., 2010; Laemmle et al., 2012). Under hypoxia SIRT1 deacylates HIF1α however, such reaction in some cases decreases HIF1α activity, whereas in other increases HIF1α activity. Obviously, more data must by accumulated on different cell lines, tissue and *in vivo* models before the real function of SIRT1 on HIF1α can be delineated. Moreover, it is also possible that SIRT1 action of HIF1α differs in different tissues and organs.

*SIRT6* is another nuclear sirtuin that controls both HIF1α and NFkB acetylation status and transcriptional activity. In the case of HIF1α, SIRT6 functions as a corepressor of HIF1α transcriptional activity, deacetylating histone 3 lysine 8 (H3K9) at HIF1α target gene promoters. In fact, regulation of glucose flux by SIRT6 appears critical because SIRT6 deficiency causes a lethal hypoglycemia (Zhong et al., 2010). Interestingly, a similar mechanism is used by SIRT6 to inhibit NFkB function. Also in this case SIRT6 deacetylates H3K9 on the promoter of selected NFkB target genes thereby decreasing accessibility to NFkB to these promoters (Kawahara et al., 2009). Importantly, in SIRT1 deficient mice, SIRT6 has shown a compensatory effect by attenuating the increased NFkB activity due to an increased acetylation state (Schug et al., 2010). In conclusion both SIRT1 and SIRT6, although with different mechanisms, represent negative regulators of NFkB activity.

*SIRT2* has been shown to deacetylate subunit p65 of NFkB on lysine 310 (K310) in the cytoplasm (Rothgiesser et al., 2010). In this way SIRT2 inhibits NFkB activation and transcription of NFkB target genes following TNF stimulation (Rothgiesser et al., 2010). In fact, SIRT2 silenced cells have an increased activation of NFkB and a lower percentage of cell death following TNF exposure (Rothgiesser et al., 2010). Therefore, NFkB can be deacetylated by SIRT2 in the cytosol and by SIRT1 in the nucleus.

*SIRT3* controls HIF1α activation indirectly. In fact, SIRT3 reduces mitochondrial ROS and activates cellular pathways and enzymes scavenging ROS (Finley et al., 2011; Pellegrini et al., 2012). In particular, by decreasing ROS levels, SIRT3 stabilizes HIF degrading enzyme prolyl hydroxylase (PHD) lowering HIF1α levels (Finley et al., 2011). Interestingly, SIRT3 deficiency is associated with tumor growth in xenografts and SIRT3 expression is lowered in several cancers and cancer cell lines (Finley et al., 2011).

Giving the fact that sirtuins regulates both HIF1α and NFkB and the central role that these two transcription factors have during tumor progression, the possibility to act on sirtuins in order to control HIF1α and NFkB has drawn much attention. Therefore, presently, a great deal of efforts have been put in producing Sirtuins modulators. Several natural compounds such as resveratrol, quercetin, piceatannol, and other polyphenols have been shown to modulate sirtuins function and particularly SIRT1 (Chung et al., 2010; Gertz et al., 2012). However, their action is not limited to SIRT1 but influences other enzymes such as phosphodiesterases (PDEs) and AMP kinase (AMPK; Dallas et al., 2008). An up-to-date and accurate review of inhibitors and activators of sirtuins has been recently published (Villalba and Alcaín, 2012).

## Ongoing Clinical Trials and Future Directions

### HIF1α

The recognition of the central role of HIF in tumor growth and progression and the *in vitro* and *in vivo* demonstration of tumor growth inhibition by many HIF1α inhibitors, is currently translating in clinical trials for some of them. In particular, 2ME2 is undergoing Phase II clinical trials in patients with breast, prostate, and ovarian cancer (Semenza, 2012; Xia et al., 2012). Similarly also molecules derived from 2ME2 have been selected for evaluation of Phase I clinical trials (Semenza, 2012; Xia et al., 2012). Analogs of geldanamycin (GA) are in Phase II clinical trials in patients with VHL disease, breast cancer, etc (Semenza, 2012; Xia et al., 2012). Bortezomib (velcade) has been approved in the US by FDA for use in multiple myeloma, based on the results from the Phase II trial. Two open-label, Phase III trials established the efficacy of bortezomib 1.3 mg/m^2^ (with or without dexamethasone) in patients with relapsed/refractory multiple myeloma (Semenza, 2012; Xia et al., 2012). EZN-2968, is currently in Phase I clinical trials (Semenza, 2012; Xia et al., 2012).

Finally, an important consideration is that many of HIF1α interacting drugs are in clinical cancer trials or are already approved for the treatment of cancer or other diseases (Xia et al., 2012).

### NFkB

Several classes of NFkB inhibitors are currently being tested in conjunction with chemotherapy and radiotherapy. In fact, a large number of clinical trials are testing the efficacy and specificity of rationally designed drugs that inhibit NFkB (for a specific trial in the US see the NIH service at: ClincalTrials.gov). In the case of NFkB for example the proteasome inhibitor Bortezomib (also used to inhibit HIF1α) has surprisingly limited side effects and therefore currently appears to be no reason to reject NFκB as drug target on the basis of potential adverse effects which might be induced by inhibition of this transcription factor (Wilczynski et al., 2011). Recently, in a phase II clinical trial, curcumin was found to be beneficial for patient with advanced pancreatic cancer (Wilczynski et al., 2011; Grynkiewicz and Slifirski, 2012). These are only two examples of a large number of NFkB inhibitors that are being currently tested. Hopefully, in the next few years several NFkB inhibitors that can increase the therapeutic efficiency of chemotherapy and radiotherapy will be successfully employed in treatment of cancer patients.

### Sirtuins

Considering the relatively short time that these enzymes are under investigation, very few sirtuin modulators are under trials. In fact, currently, there are no modulators that can specifically regulate a single sirtuin. However, resveratrol and a number of its derivatives have shown beneficial effects on a randomized double-blind cross-over trials in humans with effects similar to calorie restriction and activation of AMPK, SIRT1, and PGC-1α levels (Timmers et al., 2011). Inhibitors of SIRT1 and SIRT2 have been proposed for treatment of cancer but are far from clinical trials (Morris, 2012).

## Conclusion

Considering the central role of HIF1α and NFkB in metabolic reprogramming, inflammation, and cancer, and considering also the fact that both transcription factors are regulated by sirtuins, the possibility to develop more specific modulators acting on different steps of these molecular net, hold promising results toward new therapies with higher success rates.

## Conflict of Interest Statement

The authors declare that the research was conducted in the absence of any commercial or financial relationships that could be construed as a potential conflict of interest.
